# Does Basal Morning Luteinizing Hormone (bLH) Predict Central Precocious Puberty (CPP) in Girls?

**DOI:** 10.3390/medicina60030497

**Published:** 2024-03-18

**Authors:** Federico Baronio, Valentina Assirelli, Giuseppina Deiana, Randa AlQaisi, Rita Ortolano, Valeria Di Natale, Egidio Candela, Alessandra Cassio

**Affiliations:** 1 Pediatric Unit, IRCCS Azienda Ospedaliero-Universitaria di Bologna, 40138 Bologna, Italy; valentina.assirelli3@unibo.it (V.A.); rita.ortolano@aosp.bo.it (R.O.); valeria.dinatale@aosp.bo.it (V.D.N.); egidiocandela@gmail.com (E.C.); 2 Department of Medical and Surgical Sciences, University of Bologna, 40138 Bologna, Italy; alessandra.cassio@unibo.it; 3 UOC Territorial Pediatric Unit, AUSL Bologna, 40124 Bologna, Italy; deiana.giuseppina27@gmail.com; 4 Pediatric Endocrinology, Mu’tah University, Alkarak 61710, Jordan; alqaisiranda@gmail.com

**Keywords:** central precocious puberty, GnRH test, basal luteinizing hormone (bLH)

## Abstract

*Background and Objectives*: bLH is considered an excellent biochemical predictor of CPP. However, its utilization in clinical practice shows some uncertainties. This study aims to evaluate the diagnostic power of bLH and propose a diagnostic algorithm for CPP. *Materials and Methods*: We conducted a monocentric cohort retrospective study evaluating all females referred for suspicion of CPP between 1 January 2017 and 31 December 2020 who underwent a GnRH test. Auxological, hormonal, and instrumental data were collected, including pelvic ultrasonography and bone age (BA) assessment. Simple linear regression, *t*-test, and ROC tests were utilized to study the diagnostic value of basal hormone levels. Two hundred thirteen girls were included in the study. They were subdivided into two groups according to the results of the GnRH test: Group 1, with LH peak > 5 IU/L (pubertal) and 79 patients (37%), and Group 2, with an LH peak ≤ 5 IU/L (prepubertal) and 134 patients (63%). *Results*: The ROC curve showed that bLH level > 1.5 Ul/L best predicts a pubertal response to the GnRH test (AUC 0.8821, accuracy 82%), with low sensitivity (34%). The multivariate analysis found that bLH > 0.5 IU/L, basal FSH (bFSH) > 3.5 IU/L, bLH/bFSH ratio > 0.16, BA advancement > 1.7 years, uterine volume > 3.6 mL, longitudinal uterine diameter > 41 mm, and the presence of endometrial rhyme were significantly associated with a pubertal response at the GnRH test. An algorithm based on these features was created, and its application would reduce the number of GnRH tests by 34%. Overall, 96.2% of Group 1 patients reached the LH peak at the 30th minute of the GnRH test, supporting the hypothesis that the GnRH test duration could be reduced to 30 min. *Conclusions*: Morning bLH > 1.5 IU/L could be carefully used as a diagnostic predictor of CPP. The GnRH test, even reduced to 30 min, could be reserved for girls who show low intermediate morning bLH and specific clinical signs of pubertal development.

## 1. Introduction

Pubertal development in children is usually activated by the maturation of the hypothalamic–pituitary–gonadal (HPG) axis, starting with an increased pulsatility of the hypothalamic GnRH (gonadotropin-releasing hormone) secretion at night, which leads to an increase in basal luteinizing hormone (bLH). The biochemical marker of pubertal onset is classically represented by the ratio between bLH and basal follicular stimulating hormone (bFSH) > 1 [1,2,3].

Incomplete manifestations of pubertal development, such as isolated premature thelarche or slowly progressing/temporary forms of precocious puberty (PP), have been frequently reported in recent years [2,4]. Isolated premature thelarche is a benign condition consisting of the appearance of breasts before the age of 8 in girls that is characteristically not associated with further signs of pubertal development [5]. However, since precocious thelarche is the first sign of central precocious puberty (CPP), follow-up and clinical and laboratory monitoring are indicated.

Several diagnostic protocols help pediatric endocrinologists evaluate girls with suspected PP [6,7]. Typically, after a first clinical examination, they include a hand X-ray for bone age determination, a pelvic ultrasound for uterine and ovarian measures, basal serum gonadotropins, and estrogen levels. The evaluation of skeletal maturation using the comparative method of Greulich and Pyle [8] can help in the differential diagnosis between true precocious puberty and incomplete or non-progressive variants. Xu et al. [9] consider the advancement of bone age > 2 standard deviation (SD) to chronological age the most important predictive factor correlated with the risk of CPP.

There is an ongoing debate regarding the accuracy of ultrasonographic uterine features, such as diameter and volume, in indicating pubertal onset and stage. The European consensus in 2009 defined cut-off limits. The usefulness of ovarian parameters remains controversial [6,10,11].

When conclusive examinations are not obtained, a gonadotropin-releasing hormone (GnRH) test is used to confirm CPP. An LH peak above 5 IU/L and stimulated LH/FSH peak ratio > 1 are considered cut-offs to confirm CPP [2,6]. However, performing this testing procedure requires multiple blood samples within a 60 min timeframe at specialized medical facilities, making it intrusive, expensive, and time-consuming.

In recent years, alternative diagnostic techniques have been investigated to replace the GnRH test or at least shorten its usual length [12]. Among the newest laboratory data considered are levels of inhibin B, SHBG, and urinary gonadotropins. However, these tests are not easily accessible in all centers, can be expensive, and do not yet have a well-defined cut-off level [13,14,15].

In 2007, a Consensus Statement advocated using bLH levels as a diagnostic tool for CPP instead of the GnRH test [6]. However, there is no universal agreement on the baseline LH cut-off values that signal the onset of puberty, so the GnRH test is still commonly used.

The cut-off values for bLH associated with the likelihood of CPP vary among authors and range from 0.1 to 1.1 IU/L [16,17,18,19], depending on laboratory assays and cut-off sensitivities.

Pasternak et al. [17] showed that bLH levels under 0.1 IU/L can rule out CPP, while Lee et al. [17] defined bLH levels above 1.1 IU/L as diagnostic for CPP. Zang et al. [13] and Cao et al. [20] identified bLH levels of 0.58 IU/L and 0.53 IU/L, respectively, as diagnostic for CPP.

It is important to consider local laboratory data when applying these findings in clinical practice, as recommended by Harrington et al. [21]. Additional studies involving larger patient groups are required to establish a universally accepted bLH cut-off point or clinical approach.

Some authors have suggested a shorter duration of the GnRH test, as they found that LH peaks could appear at 45 or 60 min [20,22,23], but there is still no agreement.

At our center, it is standard practice to perform a GnRH test on all patients who present with early thelarche and bone age advancement of at least one year compared to their chronological age to diagnose CPP.

The main aim of this study is to retrospectively determine the bLH diagnostic threshold for CPP in a group of girls with suspected CPP by verifying it with the LH peak value of the GnRH test. Our secondary objective is to create an algorithm to help pediatric endocrinologists decide whether to use the GnRH test to diagnose CPP in suspected cases.

## 2. Materials and Methods

Design of the study: A retrospective monocentric study was conducted at the Pediatric Unit of the IRCCS Azienda Ospedaliero-Universitaria di Bologna, Policlinico di Sant’Orsola in Bologna, Italy. The study included all consecutive female patients with precocious thelarche and advanced bone age referred to our center between 1 January 2017 and 31 December 2020 who received a GnRH test for suspected CPP. The diagnostic criteria used for performing the GnRH test were based on the Consensus Criteria Guidelines [6], defined by the onset of thelarche before 8 years of age and a bone age (BA) advancement of at least 1 year. Exclusion criteria were age at first evaluation under 2 years old, isolated premature thelarche without bone age advancement, a diagnosis of peripheral precocious puberty, and isolated premature pubarche.

The clinical, biochemical, hormonal, and instrumental data were reviewed using retrospective medical reports. The height was measured using a Harpenden stadiometer (Holtain Ltd., Crosswell, UK) and the Body Mass Index (BMI) was calculated according to the formula weight (kg)/height (m^2^). The height, weight, and BMI variables were standardized using standard deviation scores (SDS) based on Italian norms [24].

The assessment of pubertal development, namely, breast development and pubic hair growth, was conducted using the Tanner criteria [25].

The bone age (BA) was determined using the Greulich and Pyle atlas [8], which involved the analysis of an X-ray image of the left hand and wrist. Bone age advancement (BA-CA) is determined by subtracting the chronological age (CA) from BA to assess skeletal maturity.

The GnRH test was conducted by administering gonadorelin acetate intravenously at a standard dose of 50 μg. The levels of LH and FSH were measured at 0, 30, and 60 min. A blood sample was also taken from all patients to evaluate 17-β estradiol. The levels of LH, FSH, and 17-β estradiol were assessed using chemiluminometric immunoassay through the utilization of the Access Immunoassay System. The LH sensitivity threshold ranged from 0.2 to 250 IU/L, while the FSH sensitivity threshold ranged from 0.2 to 200 IU/L. The limit for estradiol has changed throughout time, with a decrease in the analytical sensitivity of the technique from 15 pg/mL to 20 pg/mL. When the measured value falls below this limit, the report only indicates the lower value at the specified cut-off (<15 pg/mL or <20 pg/mL).

The patients were categorized according to the GnRH test based on the pubertal response, as defined by European guidelines [6].

Each girl suspected of early puberty underwent a transabdominal pelvic ultrasound performed by a skilled radiologist specialized in pediatric assessments. A Convex ecotomograph with B-mode ultrasound signal and a wavelength variable between 3.5 and 5.0 MHz (Philips Medical Systems, Amsterdam, The Netherlands) was used. The subject was evaluated in a supine position with a distended bladder. Measurements of uterine dimensions, including the longitudinal diameter (ULD), transverse diameter (UTD), and anteroposterior diameter (UAPD), were obtained in millimeters (mm). Additionally, the visibility of the endometrial rhyme was assessed. The uterine volume (UV) was determined by employing the ellipsoid formula, which involves multiplying the measurements of the uterine dimensions (ULD, UDT, and UAPD) by a constant factor of 0.5233 [26].

### Statistical Analysis

Descriptive analysis was employed to examine continuous variables, namely, mean and median values, along with range and standard deviation (SD) measures. To facilitate statistical analysis, the months were represented in decimal form. The distribution analysis of cases for the key descriptive variables was undertaken using the chi-square test. The *t*-test was employed to assess the difference in means of the continuous variables of the groups chosen based on the GnRH outcome test. Receiver operating characteristic (ROC) curves were generated to analyze the variables that presented statistically significant differences between the two groups. A diagnostic test that exhibited an area under the curve (AUC) of 80% or greater was deemed satisfactory. The region bounded by the curve has a range of values within the interval of 0.5 to 1.0. The test’s discriminating power is directly proportional to the magnitude of the region beneath the curve. To ascertain the area’s significance under the ROC curve, one may consult the categorization system introduced by Swets J.A [27]. The test has a high level of accuracy, as indicated by an AUC value of 1, which signifies a flawless test. A basic linear regression analysis assessed the inverse association between clinical and laboratory variables. All *p*-values were calculated using a two-tailed test, and statistical significance was determined by considering values less than 0.05. The statistical analysis in this study was conducted using two software programs: NCSS 2020 (NCSS 2020 Statistical Software, NCSS, LLC, Kaysville, UT, USA, https://ncss.com/software/ncss, accessed on January 2023) and STATA 7.0 (StataCorp, STATA Statistical Software: Release 7.0, Stata Corporation, College Station, TX, USA, 2000).

## 3. Results

Two hundred thirteen girls with early thelarche and advanced bone age underwent the GnRH test at our center and were considered for the study. The patients were subdivided into two groups according to the LH peak: Group 1, composed of 79 patients (37%) with a pubertal response in the GnRH test, defined by an LH peak > 5 IU/L, and Group 2, consisting of 134 subjects (63%) with a prepubertal response, defined by an LH peak ≤ 5 IU/L.

### 3.1. Auxological and Radiological Findings

The GnRH test was performed at a mean age of 7.96 years ± 1.0 SD (range 2.8–9), without significant differences in age distribution between Groups 1 and 2.

No significant differences were found between the two groups in pubertal stage and auxological and radiological parameters evaluated at the time of diagnosis. A pelvic ultrasound was performed on all patients.

Auxological and instrumental parameters (mean value and standard deviation) evaluated at diagnosis are reported in Table 1 for both groups.

Through the chi-square test, the presence of the endometrial rhyme was found to be significantly greater in the pubertal group (26.09%) vs. the pre-pubertal group (8.77%) (*p* = 0.001).

### 3.2. GnRH Test and Basal Gonadotropin Evaluation

The results of the GnRH test performed at the different time points are reported in Table 2 for both groups.

Estradiol levels, measured concurrently, were undetectable for 39/79 (49.3%) patients in Group 1 and 98/131 (74.8%) in Group 2. When detectable, the mean estradiol level was 38.02 ± 16.4 pg/mL in Group 1 and 35.09 ± 23 pg/mL in Group 2, without significant differences.

There were no statistically significant differences in the dose (mcg) of gonadorelin acetate administered per kilogram between the two groups (1.75 ± 0.39 in Group 1 vs. 1.82 ± 0.49 in Group 2) (*p* = 0.38).

### 3.3. Predictive Factors of CPP

The results of the multivariate analysis show significant associations between several variables and pubertal response to the GnRH test. These variables include bLH (OR 55, *p* = 0.000), bFSH (OR 2, *p* = 0.000), bLH /bFSH ratio (OR 2107. *p* = 0.000), bone age (BA) (OR 1.4, *p* = 0.001), uterine volume (UV) (OR 1.5, *p* = 0.000), uterine longitudinal diameter (ULD) (OR 1.1, p 0.000), and the presence of endometrial rhyme (OR 3.6, *p* = 0.002).

**Figure 1:**
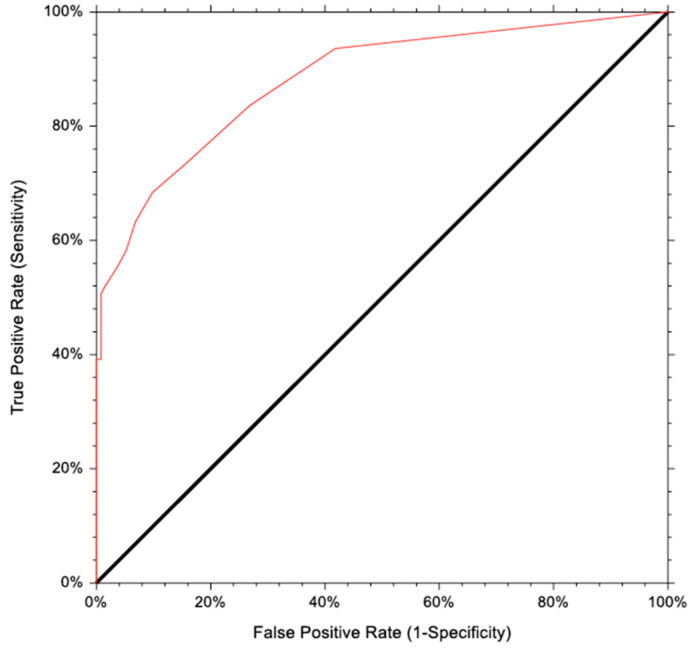
ROC curves of basal luteinizing hormone (bLH).

The analysis of the receiver operating characteristic (ROC) curve showed that the bLH level is the most precise parameter (area under the curve (AUC) of 0.8821 with an accuracy of 82%) for predicting the pubertal response in the GnRH test (Figure 1).

Basal LH > 1.5 IU/L has 100% specificity and positive predictive value for pubertal response to the GnRH test, with 34% sensitivity.

Based on the Younden index, we identified several predictive factors for pubertal response in the GnRH test. These parameters and their relative cut-off thresholds are described in Table 3.

The results of the multivariate analysis indicate that bLH > 0.5 IU/L is the only statistically significant factor associated with a pubertal response to the GnRH test, as defined by an LH peak > 5 IU/L.

Therefore, evaluating the ROC curves and the Younden index, we implemented a novel patient stratification approach (Table 4) based on the bLH level during the GnRH test.

**Figure 2:**
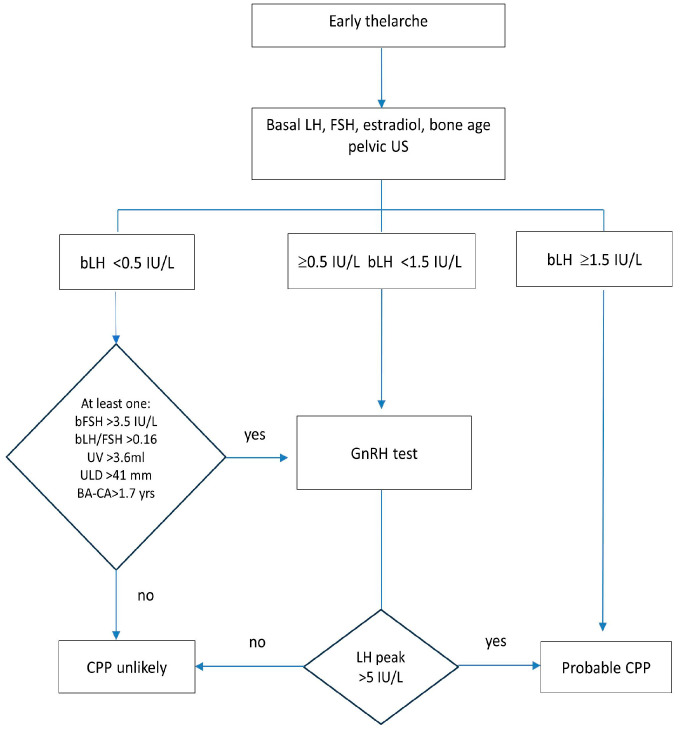
Proposed diagnostic algorithm for central precocious puberty. BA = bone age; CA = chronological age; CPP = central precocious puberty; FSH = follicle-stimulating hormone; GnRH = gonadotropin-releasing hormone; LH = luteinizing hormone; UV = uterine volume; ULD = uterine longitudinal diameter; US = ultrasound.

Based on these results, we proposed an algorithm to help diagnose CPP among patients with early thelarche and decide who needs to undergo the GnRH test (Figure 2).

Upon retrospective implementation of this diagnostic algorithm, we determined that the total number of patients who underwent the GnRH test would be reduced by 34% to 141.

### 3.4. Estimation of Peak Time in GnRH Test

We evaluated the positive predictive values of the LH and FSH stimulated peaks during the GnRH test. We found that 76 out of the 79 tests with a pubertal response reached the pubertal LH peak (>5 IU/L) by the 30th minute, indicating a positive predictive value of 96.2%. Among the three cases where the peak was observed at 60 min, the value at 30 min was around 4.9 IU/L, indicating a borderline level.

**Figure 3:**
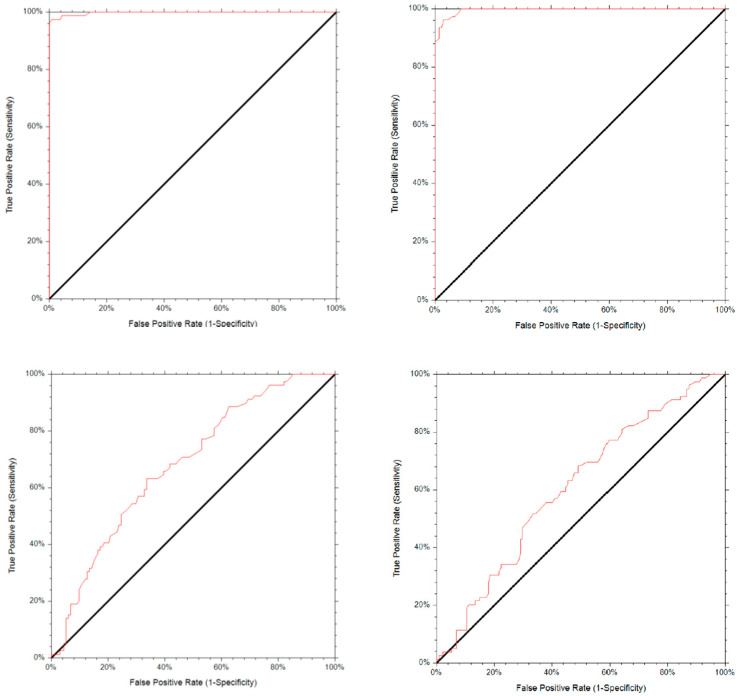
ROC curves for LH at two time points: 30 min (**top left**) and 60 min (**top right**); ROC curves for FSH at 30 min (**bottom left**) and at 60 min (**bottom right**).

To validate the reliability of conducting the GnRH test within a 30 min timeframe, ROC curves were generated for LH and FSH at both 30 and 60 min intervals (Figure 3). LH at 30 min shows higher sensitivity and specificity than LH at 60 min (97% vs. 96% and 99 vs. 97%, respectively). For FSH, we found a higher sensitivity at 60 min (68% vs. 63%) and a higher specificity at 30 min (66% vs. 51%). The ROC curves for LH and FSH at these time points are show in Figure 3.

## 4. Discussion

According to European consensus and Italian clinical practice [6,28], the GnRH test is considered the diagnostic gold standard for CPP. However, it is invasive, time-consuming, and only available in specialized centers. Several studies have been conducted in recent years to identify diagnostic biomarkers for CPP. However, none of these have found a highly sensitive and specific parameter universally accepted and used in clinical practice [17,18,20,21,29].

In our tertiary center, it is a standard practice to perform a GnRH test on girls with premature thelarche and bone age advancement to exclude CPP. Our study retrospectively evaluated a cohort of consecutive girls with premature thelarche and BA advancement who were subjected to the GnRH test for suspicion of CPP. We aimed to identify the bLH level cut-off that discriminates between pubertal and prepubertal response in the GnRH test.

We did not find a reliable cut-off of bLH that can diagnose CPP with high sensitivity and specificity. Pasternak et al. [16] found that a low bLH (≤0.1 IU/L) was sufficient to rule out CPP in 94.7% of prepubertal girls, but the sensitivity was only 64%. Lee et al. [17] reported that basal LH > 0.1 IU/L and LH/FSH ratio > 0.04 IU/L predicted positive responses to the GnRH test. The LH/FSH ratio was the best parameter for predicting positive results in the GnRH test, with a sensitivity of 54.4% and specificity of 93.7%. Durà-Travè et al. [7] found that a bLH threshold > 1.0 UI/L shows 100% positive predictive value for CPP, but no convincing clinical features can predict a positive response to the GnRH test.

In our cohort, we found a higher threshold for bLH (>1.5 IU/L), with a positive predictive value of 100% for CPP, as already reported by Durà-Travè [7], but with low sensitivity (34%). Although the study design and conclusions are similar, our cut-offs are discordant from those reported by Durà-Travè [7]. According to our research, bLH values lower than 0.2 do not allow us to completely rule out a pubertal response to the GnRH test. Due to the sensitivity threshold, we could not evaluate the difference in bLH levels between 0.1 and 0.2 UI/L. Therefore, it cannot be excluded that even in our sample, a bLH threshold lower than 0.1 can certainly exclude the condition of CPP due to the limited sensitivity of our laboratory. Using a bLH threshold of >0.5 IU/L as the best cut-off, determined by ROC curve analysis, would generate an unacceptable number of false positives (19%) with 90% specificity and 68% sensitivity. Additionally, among patients with a bLH level < 0.5 IU/L, 17% of cases displayed a stimulated LH peak > 5 IU/L. This clinical–laboratory discrepancy may depend on whether these patients were analyzed early in HPG axis activation when the LH pulsatility is still intermittent [16,17,19,28].

Basal LH levels can be a useful indicator of pubertal development in cases with high bLH levels. However, our data clearly show that a GnRH test is necessary to confirm CPP in girls with intermediate and even low basal LH levels if there are clinical signs of puberty present. This is in line with the findings of Lee DS et al. [17].

It is common to include bone age assessment in the diagnostic workup for precocious puberty in girls [29]. In addition, pelvic ultrasonography can be extremely helpful in determining uterine volumes and diameters or excluding peripheral causes of pubertal development.

The multivariate analysis found that some variables are significantly associated with a pubertal response to the GnRH test and could be considered predictive factors for CPP. These are represented by bFSH levels, the bLH/bFSH ratio, BA advancement, uterine volume (UV), uterine longitudinal diameter (ULD), and endometrial rhyme. Our study agrees with other authors regarding estradiol levels [30]. Although it is undetectable in a higher percentage of patients from the prepubertal group, the detectable estradiol level does not discriminate between the two groups. In our cohort, patients with LH peak > 5 IU/L (pubertal group) showed significantly higher ULD, UV, presence of endometrial rhyme, BA advancement, and levels of gonadotropins at each time of the test, along with a higher basal LH/FSH ratio (Table 1 and Table 2). Table 3 shows the most accurate cut-off values for each variable that can predict a pubertal GnRH test result using ROC curves and the Youden index.

It is not surprising that there is a relationship between the advancement of bone age and the size or longitudinal diameter of the uterus in girls going through puberty. Several authors have reported this correlation in recent decades [29]. However, in some cases, multiple evaluations, often combined, of these features are necessary. This is especially true when there are no clear clinical signs of pubertal progression.

We proposed a diagnostic approach that combines biochemical results with clinical and radiological features, such as bone age advancement and uterine parameters, to better identify girls with low or intermediate bLH levels who require further evaluation through a GnRH test. Our algorithm included the best parameters significantly correlated with pubertal response, identified by LH peak > 5 IU/L. However, it is important to note that the accuracy of bone age (BA) and uterine ultrasound evaluation depends on the operators’ skills and should be considered cautiously. In our study, BA and uterine ultrasound parameters were examined by experienced radiologists in the same facility.

By applying the algorithm presented in Figure 2, we can decrease the number of GnRH tests by 34%. This can be achieved by excluding girls with baseline LH values greater than 1.5 U/L and patients with baseline LH values less than 0.5, provided no other predictive factors exist. Harrington et al. developed a diagnostic algorithm that used a bLH cut-off of ≥0.3 IU/L to reduce the use of the GnRH test by 75% [21]. This did not decrease the diagnostic rate of CPP. However, in our cohort, a similar cut-off would not be effective in distinguishing between prepubertal and pubertal girls. Therefore, we developed a different algorithm.

Our data showed that the GnRH test could be shortened to 30 min. In our cohort, 96% of pubertal girls exhibited the LH peak at the 30 min mark, and the ROC curve analysis verified this outcome. This aligns with recent studies that propose shortening the duration of the GnRH test [20,22,28,30]. In the research conducted by Kim et al., it was found that the LH peaks were achieved 30 min after the GnRH stimulation test in the CPP group and 45 min after the stimulation in the early thelarche group. However, after performing an ROC curve analysis, they concluded that the 45 min samples were the most accurate for diagnosing CPP [22]. Our research supports the findings of Pellegrin et al., who discovered that a 30 min GnRH test has a high sensitivity rate of 99.03% and specificity in diagnosing CPP in females. Additionally, two samples collected after 30 and 60 min, respectively, provide 100% sensitivity and specificity in diagnosing CPP in both males and females [2,31].

One limitation of our study is represented by the analytical sensitivity of the method used at our center, which reaches a minimum value of 0.2 IU/L. At the same time, numerous studies identify the optimal cut-off as 0.1 IU/L [1]. Moreover, it is worth noting that our study has some limitations related to its retrospective nature and the sample size. However, one of its strengths is that all pharmacological tests and clinical and radiologic evaluations were conducted in the same center, which helped minimize bias due to subjective evaluation.

At our center, we conduct the GnRH test using a fixed administration of 50 mcg of gonadorelin acetate. Although we cannot rule out the possibility of insufficient stimulation, we have observed that in girls with an LH peak greater than 5 IU/L, the mean dosage of gonadorelin per body weight was lower than in others [31].

## 5. Conclusions

In conclusion, the measurement of bLH is a reliable parameter that should be included in the initial diagnostic workup of patients with suspected CPP. However, in certain cases, the confirmation of CPP requires a GnRH test, which can be reduced to 30 min. Using the proposed diagnostic algorithm could help reduce the time and costs associated with the diagnosis of CPP on a case-by-case basis. However, further prospective studies are necessary to validate its effectiveness.

## Figures and Tables

**Table 1:** Auxological and radiological findings (mean ± SD) in the two groups of patients.

	Group 1 (Pubertal) n. 79	Group 2 (Prepubertal) n. 134	*p*-Value
Age (years)	7.97 ± 1.00	7.49 ± 1.22	0.0036
Height (SDS)	0.88 ± 1.12	0.94 ± 3.33	0.76
BMI (SDS)	0.03 ± 0.81	0.30 ± 1.05	0.06
ULD (mm)	42.8 ± 6.70	37.7 ± 5.64	<0.001
UV (mL)	5.55 ± 3.46	3.12 ± 1.84	<0.001
BA (years)	9.39 ± 1.54	8.51 ± 1.66	<0.001
BA advancement (years)	1.66 ± 1.02	1.36 ± 1.36	0.09

BMI = Body Mass Index; ULD: uterine longitudinal diameter; UV: uterine volume; BA: bone age.

**Table 2:** Basal and stimulated gonadotropin levels (mean ± SD) after GnRH test in Groups 1 and 2.

	Group 1 (Pubertal) n. 79	Group 2 (Prepubertal) n. 134	*p*-Value
Basal FSH (IU/L)	4.90 ± 2.10	2.46 ± 1.56	<0.001
FSH (IU/L) 30′	11.85 ± 6.32	9.25 ± 4.78	0.0019
FSH (IU/L) 60′	13.74 ± 7.70	11.8 ± 6.09	0.0433
Basal LH (IU/L)	1.33 ± 1.22	0.21 ± 0.20	<0.001
LH (IU/L) 30′	14.47 ± 11.6	2.29 ± 1.19	<0.001
LH (IU/L) 60′	12.12 ± 8.70	2.25 ± 1.14	<0.001
Basal LH/FSH(IU/L)	0.26 ± 0.21	0.07 ± 0.01	<0.001

**Table 3:** Factors that correlate with a pubertal response in GnRH test and relative cut-off.

Predictive Factors	Cut-Off	Sensitivity	Specificity
bLH	>0.5 IU/L	68%	90%
bFSH	>3.5 IU/L	75%	79%
bLH/bFSH	>0.16	62%	81%
UV	>3.6 mL	68%	72%
ULD	>41 mm	64%	72%
BA advancement	>1.76 years	81%	69%

UV = uterine volume; ULD = uterine longitudinal diameter; BA = bone age.

**Table 4:** Patient stratification according to their bLH and predictive factors.

		Group 1 (LH Peak > 5 IU/L) n. pts	Group 2 (LH Peak ≤ 5 IU/L) n. pts	Total n. pts
bLH ≥ 1.5 IU/L		31	0	31
bLH ≥ 0.5 < 1.5 IU/L		23	13	36
bLH < 0.5 IU/L	n.PF ≥ 1	25	80	105
	No PF	0	41	41
Total n.pts		79	134	213

LH = luteinizing hormone; PF = predictive factors; pts = patients.

## Data Availability

All clinical data and materials are available in our Pediatric Unit.

## Abbreviations

AUC	Area Under the Curve
bFSH	Basal Follicular Stimulating Hormone
bLH	Basal Luteinizing Hormone
BA	Bone Age
CPP	Central Precocious Puberty
CA	Chronological Age
GnRH	Gonadotropin-Releasing Hormone
HPG	Hypothalamic–Pituitary–Gonadal
Mm	Millimeters
PF	Predictive Factor
PP	Precocious Puberty
PT	Premature Thelarche
ROC	Receiver Operating Characteristic
SD	Standard Deviation
US	Ultrasound
UAPD	Uterine Antero-Posterior Diameter
ULD	Uterine Longitudinal Diameter
UTD	Uterine Transverse Diameter
UV	Uterine Volume
